# Exosomes from human induced pluripotent stem cells-derived keratinocytes accelerate burn wound healing through miR-762 mediated promotion of keratinocytes and endothelial cells migration

**DOI:** 10.1186/s12951-022-01504-8

**Published:** 2022-06-21

**Authors:** Yunyao Bo, Lijun Yang, Baiting Liu, Guiping Tian, Chenxi Li, Lin Zhang, Yuan Yan

**Affiliations:** 1 Department of Histology and Embryology, School of Basic Medical Science, Southern Medical University, Guangzhou, 510515 China; 2 Guangdong Provincial Key Laboratory of Construction and Detection in Tissue Engineering, Guangzhou, 510515 China; 3 NMPA Key Laboratory for Safety Evaluation of Cosmetics, Guangzhou, 510515 China

**Keywords:** Exosome, Burn, Induced pluripotent stem cells, Keratinocytes, Endothelial cell

## Abstract

**Background:**

The use of keratinocytes derived from induced pluripotent stem cells (iPSCs-KCs) may represent a novel cell therapy strategy for burn treatment. There is growing evidence that extracellular vesicles, including exosomes, are primary mediators of the benefits of stem cell therapy. Herein, we thus explored the effects of exosomes produced by iPSCs-derived keratinocytes (iPSCs-KCs-Exos) in a model of deep second-degree burn wound healing and evaluated the mechanistic basis for the observed activity.

**Methods:**

iPSCs-KCs-Exos were isolated from conditioned medium of iPSCs-KCs and verified by electron micrograph and size distribution. Next, iPSCs-KCs-Exos were injected subcutaneously around wound sites, and its efficacy was evaluated by measuring wound closure areas, histological examination, and immunohistochemistry staining. The effects of iPSCs-KCs-Exos on proliferation and migration of keratinocytes and endothelial cells in vitro were assessed by EdU staining, wound healing assays, and transwell assay. Then, high-throughput microRNA sequencing was used to explore the underlying mechanisms. We assessed the roles of miR-762 in iPSCs-KCs-Exos-induced regulation of keratinocytes and endothelial cells migration. Furthermore, the target gene which mediated the biological effects of miR-762 in keratinocytes and endothelial cells was also been detected.

**Results:**

The analysis revealed that iPSCs-KCs-Exos application to the burn wound drove the acceleration of wound closure, with more robust angiogenesis and re-epithelialization being evident. Such iPSCs-KCs-Exos treatment effectively enhanced endothelial cell and keratinocyte migration i*n vitro.* Moreover, the enrichment of miR-762 was detected in iPSCs-KCs-Exos and was found to target promyelocytic leukemia (PML) as a means of regulating cell migration through a mechanism tie to integrin beta1 (ITGB1).

**Conclusion:**

These results thus provide a foundation for the further study of iPSCs-KCs-Exos as novel cell-free treatments for deep second-degree burns.

**Graphical Abstract:**

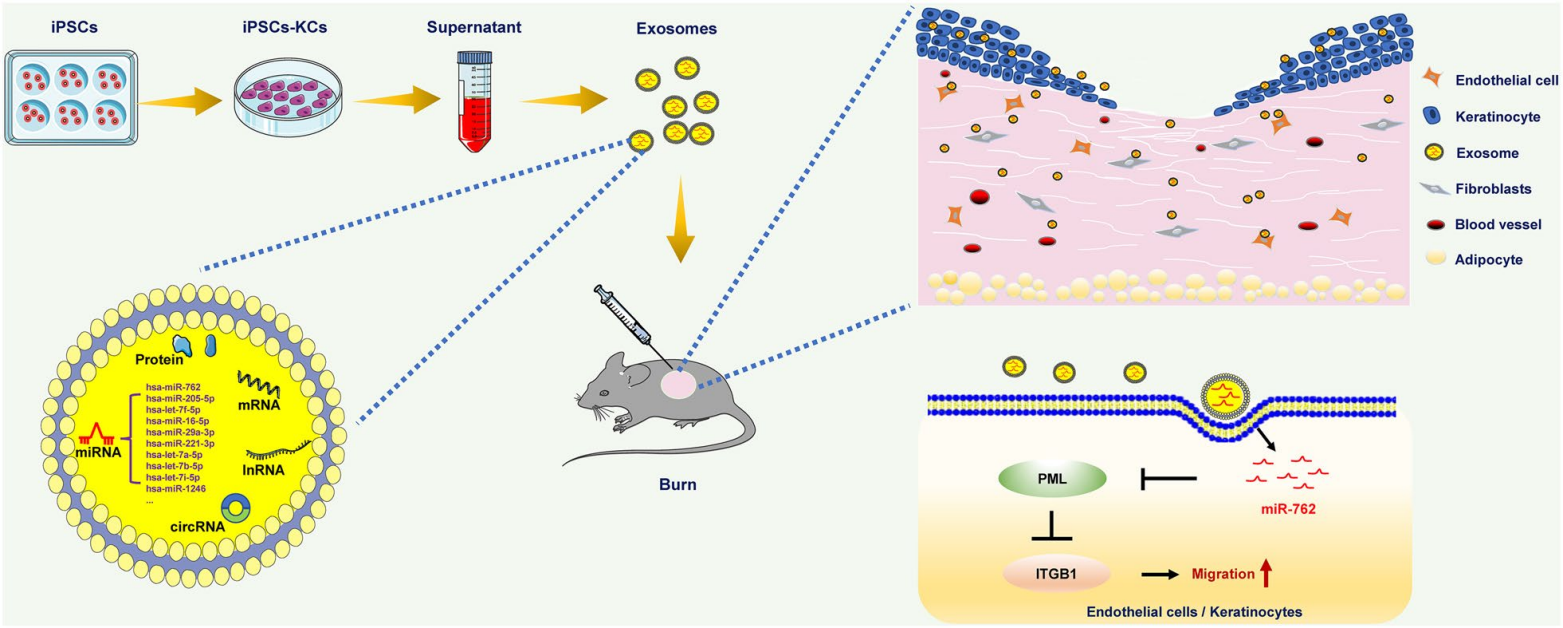

## Introduction

Burns is a form of serious traumatic injury that causes substantial global morbidity, and there is thus an urgent need to define novel approaches to expediting burn healing [1–3]. Preclinical and clinical evidence suggests that keratinocyte-based therapies offer great promise in the context of burn management [4–6]. However, there are substantial challenges to large-scale keratinocyte preparation efforts because donor keratinocyte yields are often very limited. The ability of these donated cells to proliferate and differentiate in vitro is also highly variable. The Yamanaka lab first developed induced pluripotent stem cells (iPSCs) and highlighted their potential as a novel source of many cellular lineages for use in therapeutic contexts [7–9]. The generated iPSCs are extremely similar to embryonic stem cells being capable of generating all cell lineages of different tissues including skin, highlighting a novel method of improving cell-based wound treatment efforts while minimizing ethical concerns and the risk of immune-mediated graft rejection [10, 11].

We have previously shown that iPSCs-derived keratinocytes (iPSCs-KCs) hold great promise for treating wound healing [12, 13]. However, the safety profile of these cells is not known and they are potentially limited by the risk of immunogenicity and genetic instability. Recent work has suggested that small membrane-bound exosomes derived from cells can facilitate a substantial fraction of the paracrine signaling necessary to facilitate intracellular communication [14, 15]. Exosomes of stem cells including bone marrow mesenchymal stem cells, human adipose stem cells, human amniotic epithelial cells, and iPSCs can play an anti-inflammatory and anti-fibrosis role, inhibit oxidative stress and enhance angiogenesis, thus promoting skin wound healing [16–20]. As such, we speculate that iPSCs-KCs-derived exosomes (iPSCs-KCs-Eoxs) have the potential to play a direct role in the facilitation of burn wound healing. Up to now, there are no related studies on the iPSCs-KCs-Exos, and there are few studies on the primary keratinocyte’s exosomes in skin wound healing [21–23].

Herein, we utilized iPSCs-KCs-Exos to treat murine skin wounds to test the therapeutic efficacy of these cell-free vesicles. We found that such iPSCs-KCs-Exos treatment was conducive to more rapid wound closure as well as enhanced endothelial cell and keratinocyte migration. We then evaluated the microRNAs present within these exosomes to identify important mediators of this wound healing phenotype and found miR-762 to be highly enriched in iPSCs-KCs-Exos and to serve as an important mediator of cellular migration owing to its ability to target PML. Overall, these data highlight the important roles played by iPSCs-KCs-Exos and miR-762 in the treatment of cutaneous wounds.

## Materials and methods

### Cell culture

The human iPS cell line was provided by Stem Cell Bank, the Chinese Academy of Sciences. hiPSCs were cultured on Matrigel (BD Biosciences, CA) in mTeSR^TM^1 medium (Stemcell Technologies, CA) and passaged every 4–7 days. hiPSCs were induced to keratinocytes as described previously [11]. Briefly, hiPSCs were switched to unconditioned medium (UM) supplemented with retinoic acid (RA): DMEM/F12 containing 20% knockout serum replacer, 1 mM L-glutamine, 1 × MEM nonessential amino acids, 0.1 mM β-mercaptoethanol (all from Life Technologies, USA) and 1 mM all-trans RA (Sigma-Aldrich, USA). Cells were cultured in UM + RA for 7 days, changing medium daily. On day 8, cells were passaged onto gelatin-coated plates in Keratinocyte Defined Serum-free Medium (K-DSFM, Life Technologies). After 4 weeks, differentiated cells were subcultured using trypsin to obtain high purity keratinocytes populations. Terminal differentiation was induced by adding 1.5 mM CaCl_2_ in K-DSFM for 10 days. HaCaT cells and human umbilical vein endothelial cells (both from COBIOER, China) were cultured in Dulbecco's modified Eagle’s medium (DMEM, Gibco, USA), supplemented with 10% fetal bovine serum (Gibico, USA).

### Extraction and identification of iPSCs-KCs-Exos

iPSCs-KCs were cultured in K-DSFM for 48 h. After collecting the supernatant, exosomes were isolated and purified by serial ultracentrifugation, using ultracentrifuge (Beckman, Germany). Briefly, the supernatant was centrifuged at 300 ×*g* for 10 min, 2000 ×*g* for 10 min, then 10,000 ×*g* for 30 min at 4 ℃ to remove cells and debris. The supernatants were then centrifuged at 100,000 ×*g* for 70 min at 4 ℃. Applying PBS to resuspend the pellet and centrifuged again at 100,000 ×*g* for 70 min. Finally, the exosomes were re-suspended in PBS. The concentration of exosomes was measured by Enhanced BCA Protein Assay Kit (Beyotime, China). Then, transmission electron microscopy (TEM) was used to observe the morphological structure of exosomes. Size distribution and concentration of iPSCs-KCs-Exos were analyzed by nanoparticle tracking analysis (NTA) using Nanosight LM 10 (Malvern Panalytical, UK).

### Treatment of deep second-degree burns

The 8-week-old C57BL/6 mice were purchased from the Experimental Animal Center of Southern Medical University. After the hair removal from the back of the mice, the opening at one end of a cylindrical plastic tube with a diameter of 1.5 cm was affixed to the back skin of the mice. Then pour boiling water into the tube at a height of 2 cm and then the tube was removed after 18 s. After burning, local subcutaneous injections of PBS or exosomes were given to the wound site.

### Histological observations

The skin tissues were fixed with 4% paraformaldehyde for 72 h, embedded in paraffin, and then cut into 5 mm thick sections. The sections were stained with hematoxylin–eosin (H&E) staining (Leagene, China) and Masson's tri-chrome staining (MXB Biotechnologies, China). The re-epithelialization was calculated in the accordance with the formula [a distance of the re-epithelium]/ [a distance of the wound] × 100.

### Immunohistochemical assay

Tissue sections for immunohistochemical staining were dewaxed, incubated with 3% H_2_O_2_, and heated with citrate buffer (pH 6.0). After being blocked by goat serum, sections were incubated overnight with CD31 primary antibody (Abcam, UK) at 4 °C. The sections were then incubated for 1 h with biotinylated secondary antibodies (Zhong Shan Golden Bridge Biotechnology, China). Peroxidase activity was detected by diaminobenzidine (DAB). Finally, sections were counterstained with hematoxylin.

### Immunofluorescence assay

At room temperature, cells were fixed with 4% paraformaldehyde for 30 min and then drilled with 0.5% Triton X-100 for 15 min. The cells were incubated overnight at 4 °C with primary antibodies against K14, loricrin, or involucrin (Proteintech, USA) and then incubated with Alexa Fluor 647-conjugated sheep anti-rabbit second antibody (Abcam, UK) for 1 h at room temperature. DAPI (500 ng/mL) was used to stain nuclei. Fluorescence was observed under an inverted fluorescence microscope (Leica, Germany).

### EDU staining

After cells were treated for 48 h, EdU was added to the culture medium and incubated for 4 h. The cells were fixed with 4% paraformaldehyde and then drilled with 0.5% Triton X-100 for 15 min. Then, the cells were stained using Beyoclick™ EDU cell proliferation kit Alexa Fluor 488 (Beyotime, China) and imaged using an inverted fluorescence microscope (Leica, Germany).

### Wound healing assay

Cells were seeded into 24-well plates and cultured overnight. After each well was filled with 95% monolayer of cells, the tip of the 200 μL pipette was used to form linear scratch wounds. At 0 h and 24 h, the wound healing was recorded by a microscope (Leica, Germany). Image J software was used to quantitatively evaluate the wound healing rate.

### Transwell assay

200 μL cell suspension (serum-free DMEM) was added to the upper chamber of Transwell, and 600 μL DMEM containing 2% FBS was added to the lower chamber. After 24 h incubation at 5% CO_2_ and 37 ℃, the cells were fixed with 4% paraformaldehyde. The cells at the lower side of the membrane were stained with Crystal Violet Staining Solution (Beyotime China) for 15 min. An inverted microscope (Leica, Germany) randomly collected 10 images from each sample.

### miRNA microarray analysis

miRNA microarray analysis was performed by Epibiotek (Guangzhou, China) as previously described [19].

### Western blot analysis

The Western blot assay was performed as previously described [19]. Antibodies used were described as follows: anti-PML, anti-ITGB1or anti β-actin (all from Abcam, UK).

### Reverse transcription-quantitative polymerase chain (qRT–PCR)

Trizol™ reagent (Invitrogen, USA) was used to extract the total RNA from cells, according to the manufacturer’s instructions. RNA was reverse-transcribed using RevertAid First Strand cDNA Synthesis Kit (Thermo Scientific, USA). qRT–PCR analysis was using ACEQ Universal SYBR qPCR Master Mix (Vazyme, China). Midtect A TrackTM miRNA qRT–PCR Starter Kit (RiboBio, China) was used for the synthesis of miRNA cDNA. Then, Midtect A TrackTM miRNA qRT–PCR Starter Kit (RiboBio, China) was used for qRT–PCR.

### Luciferase report assay

The wild type and mutant miR-762 binding sites of PML 3′UTR sequence were subcloned into psiCHECK-2 vector. MiR-762 mimics or negative control mimics were co-transfected with recombinant plasmid into HEK-293 T cells using Lipofectamine 2000 (Invitrogen, USA). According to the manufacturer’s instructions, the Firefly and Renilla luciferase activities were detected by Dual Luciferase Reporter Gene Assay Kit (Beyotime, China).

### Statistical analysis

Data were presented as mean ± standard deviation (SD) of at least three independent experiments (*n* ≥ 3). Statistical analysis was performed by independent samples *t*-test for comparison between two groups or one-way ANOVA among the groups. *p* < 0.05 was considered statistically significant.

## Results

### Characterization of iPSCs-KCs and iPSCs-KCs-Exos

**Fig. 1:**
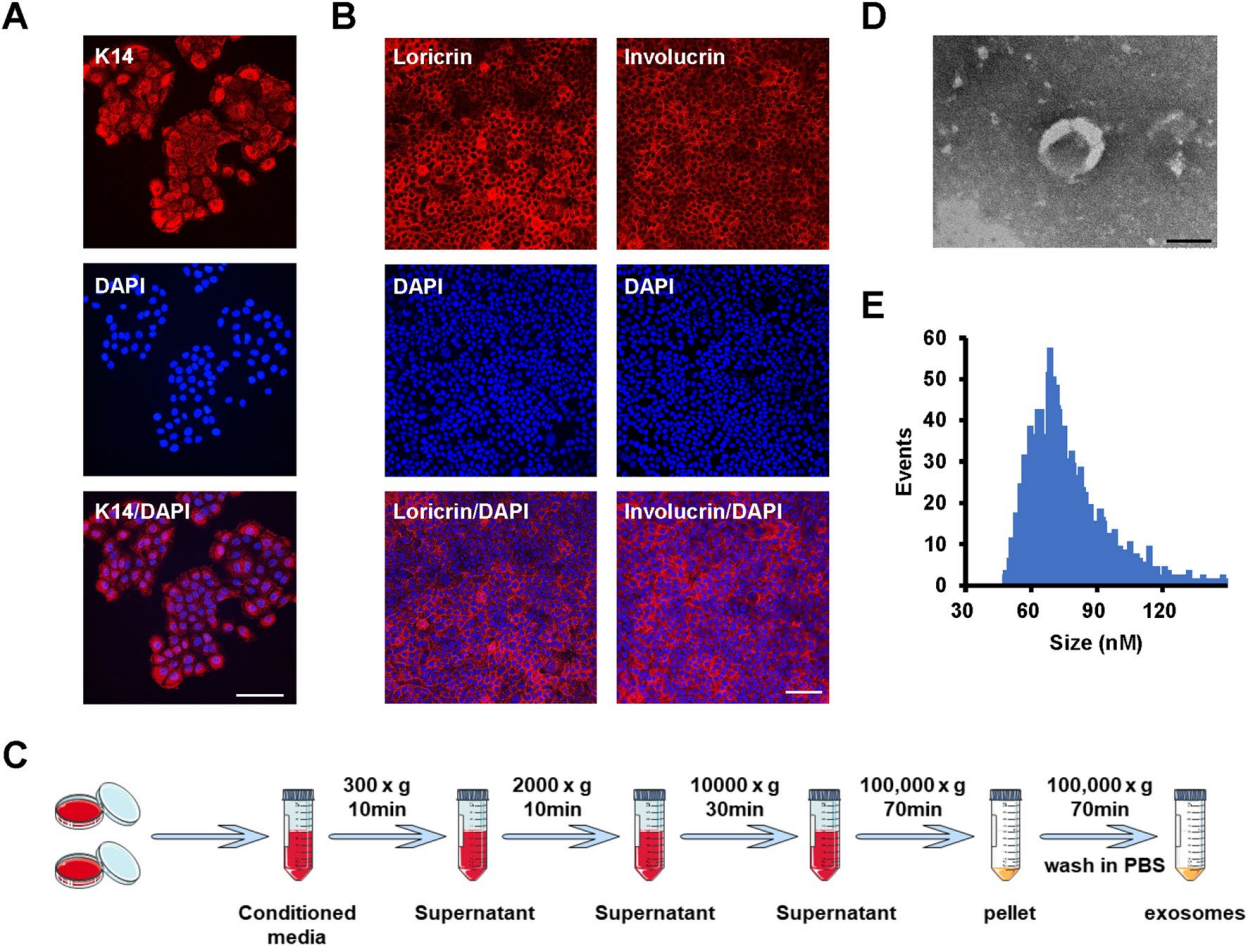
Characterization of iPSCs-KCs and iPSCs-KCs-Exos. **A** Representative images of immunohistochemical staining for K14 in iPSCs-KCs. Scale bar, 100 μm. **B** Representative images of immunohistochemical staining for involucrin and loricrin in iPSCs-KCs culture for 10 days in DKSFM supplemented with 1.5 mmol/L of CaCl_2_. Scale bar, 100 μm. Nuclei were stained by DAPI (blue). **C** Schematic diagram of iPSCs-KCs-Exos extraction process. **D** The transmission electron microscopy (TEM) image of iPSCs-KCs-Exos. Scale bar, 100 nm. **E** Nanoparticle tracking analysis (NTA) result of iPSCs-KCs-Exos

To characterize prepared iPSCs-KCs, we conducted immunohistochemical (IHC) staining for cytokeratin-14 (K14), which is a keratin marker confirming the commitment of the ectoderm to a keratinocyte fate. As shown in Fig. 1A, K14 was detectable in iPSCs-KCs. In addition, these K14^+^ cells expressed involucrin and loricrin (Fig. 1B), markers of terminal epidermal differentiation, after induced differentiation in a high calcium medium (1.5 mM CaCl_2_), indicating that these iPSCs-KCs have the ability of epidermal differentiation.

Next, iPSCs-KCs-Exos preparations were obtained by ultracentrifuging iPSCs-KCs culture supernatants (Fig. 1C) and analyzing them via transmission electron microscopy (TEM), revealing the presence of exosomes with the expected cup-shaped morphology (Fig. 1D). Nanoparticle tracking analysis (NTA) revealed these particles to have an average diameter of 75 nm (Fig. 1E). All these data suggested that these nanoparticles were actually exosomes.

### iPSCs-KCs-Exos treatment accelerates the healing of deep second-degree burns in mice

**Fig. 2:**
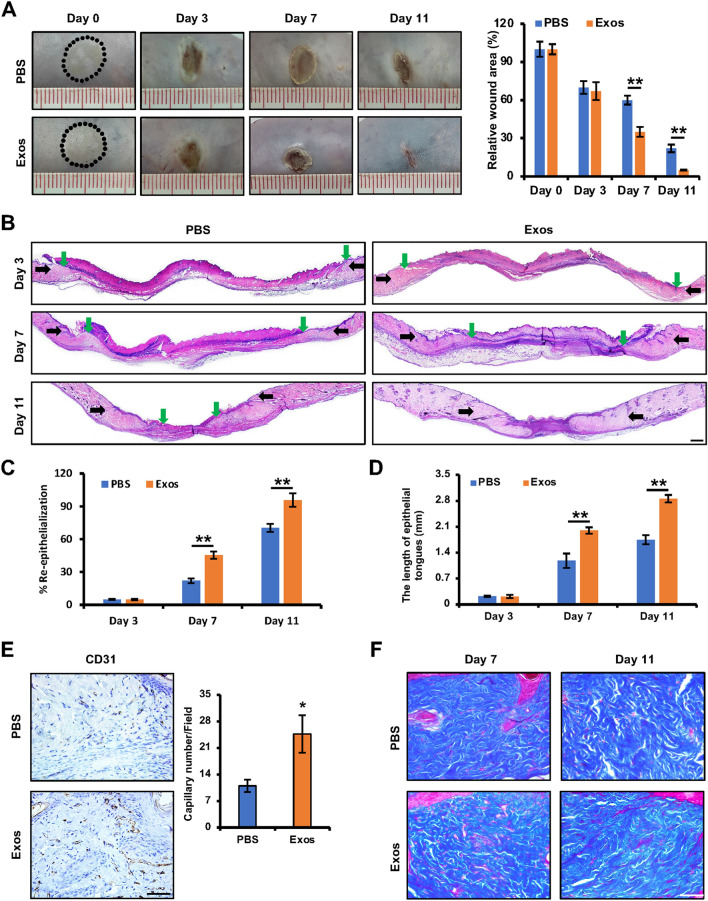
iPSCs-KCs-Exos accelerate deep second-degree burn wound healing in vivo. **A** Representative images of wounds treated with PBS or iPSCs-KCs-Exos on days 0, 3, 7, and 11 after burning and quantitative analysis of wound area in each group. n = 6 mice per group. **B** H&E staining of wounded skin sections treated with PBS or iPSCs-KCs-Exos on days 3, 7, and 11 after burning. Black arrows indicate the dermal border; green arrows indicate the epidermal margin. Scale bar, 400 μm. **C** Quantitative analysis of the re-epithelialization ratio of wounds in each group. **D** Quantitative analysis of the length of epithelial tongues of wounds in each group. **E** Immunohistochemistry staining for CD31 wounds and quantitative analysis of the numbers of stained capillaries in each group on days 11 after burning. Scale bar, 50 μm. **F** Masson’s trichrome staining of wounds in each group on days 7 and 11 after burning. Scale bar, 50 μm. *P < 0.05, **P < 0.01

To test the effects of iPSCs-KCs-Exos treatment on the healing of burn wounds, deep second-degree burns were generated in the abdominal skin of model mice, after which iPSCs-KCs-Exos or an equivalent volume of PBS was locally injected into these areas. Beginning on day 7 post-wounding, significant decreases in wound area in the iPSCs-KCs-Exos group were evident relative to the PBS group, and these improvements grew through day 11 (Fig. 2A). This suggests that iPSCs-KCs-Exos can promote the acceleration of cutaneous wound repair in vivo*.*

We additionally conducted histological analyses of these healing wounds. Improved re-epithelialization of the wound site was observed in the iPSCs-KCs-Exos treatment group on days 7 and 11 after wounding as compared to the control group when H&E staining was conducted (Fig. 2B, C). Furthermore, there was a significant increase in the length of epithelial tongues on days 7 and 11 in the iPSCs-KCs-Exos group compared to the PBS group (Fig. 2B, D). Immunohistochemical staining for CD31 further revealed that significant improvements in capillary density were evident in the iPSCs-KCs-Exos-treated wounds. (Fig. 2E). However, no differences in collagen deposition were observed when comparing iPSCs-KCs-Exos and PBS groups (Fig. 2F). Together, these data revealed a role for iPSCs-KCs-Exos as drivers of re-epithelialization and angiogenesis, thereby enabling them to accelerate the wound healing process.

### iPSCs-KCs-Exos promote the migration of keratinocytes and endothelial cells in vitro

**Fig. 3:**
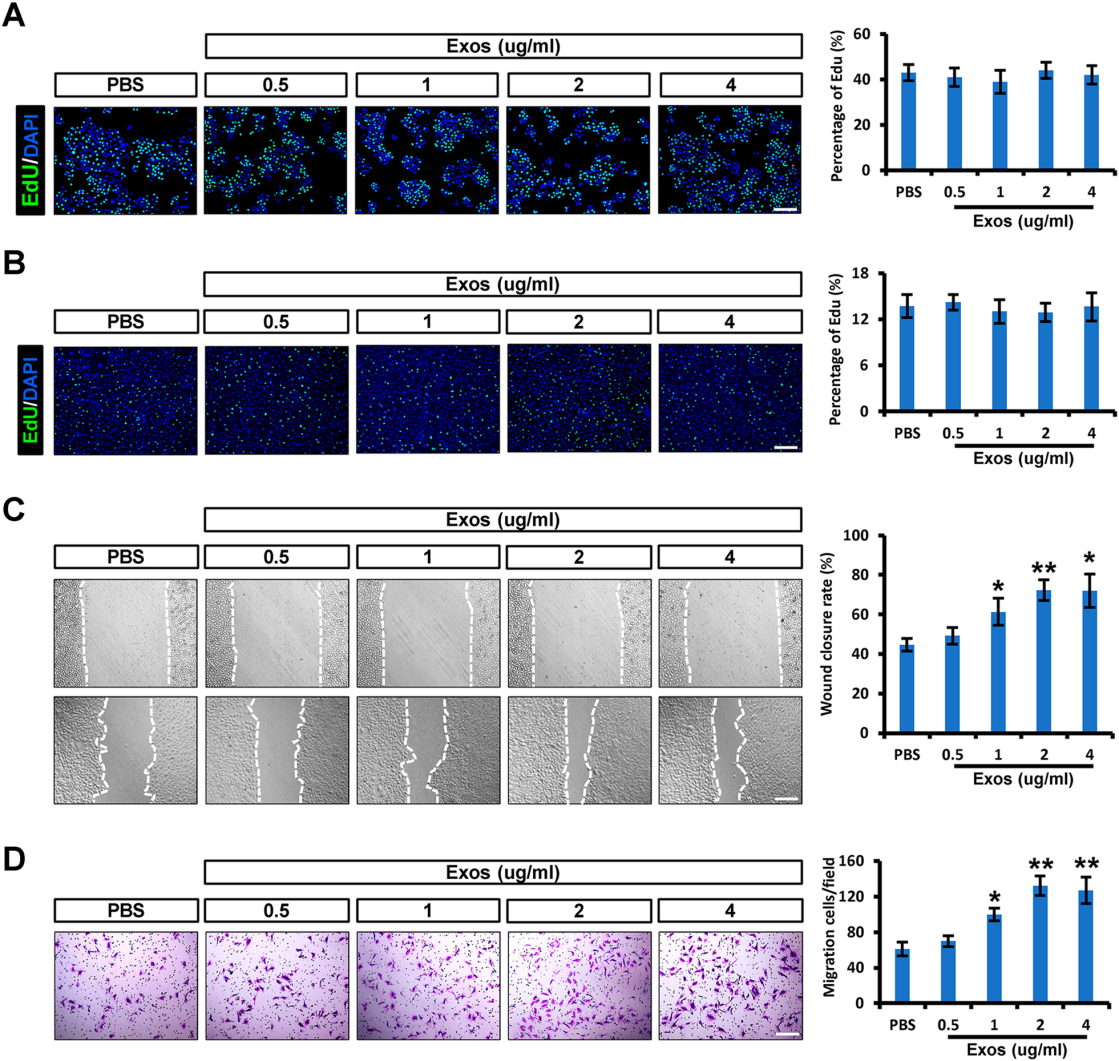
iPSCs-KCs-Exos promote the migration of keratinocytes and endothelial cells in vitro. **A** Representative fluorescence images of EdU staining of HaCaT cells treated with PBS or iPSCs-KCs-Exos (0.5, 1, 2, or 4 μg/mL) and quantitative analysis of the proliferation rates in each group. Scale bar, 200 μm. **B** Representative fluorescence images of EdU staining of HUVECs and quantitative analysis of the proliferation rates in each group. Scale bar, 200 μm. **C** Representative images of the wound healing assay and quantitative analysis of the wound healing rates in each group at 24 h. Scale bar, 200 μm. **D** Images of migrated HUVECs and quantitative analysis of the migrated cells in each group. Scale bar, 200 μm. *P < 0.05, **P < 0.01

To better elucidate the mechanisms whereby iPSCs-KCs-Exos treatment can accelerate the burn wound healing, the impact of these exosomes on healing-related cell types (endothelial cells and keratinocytes) was next assessed. EdU assays were applied to determine the effect of iPSCs-KCs-Exos on the proliferation of these two kinds of cells. HaCaT cells, a well‐established immortalized human keratinocyte cell line, and human umbilical vein endothelial cells (HUVECs) were used in this study. As shown in Fig. 3A and B, these iPSCs-KCs-Exos did not alter the proliferation of HaCaT cells or HUVECs at all doses in vitro*.* In a wound healing assay, iPSCs-KCs-Exos treatment was associated with a significantly increased wound closure rate compared to PBS at doses of 1, 2, and 4 μg/ml (Fig. 3C)*.* When evaluated using Transwell assays, HUVECs migratory activity was also found to be significantly increased following iPSCs-KCs-Exos treatment at doses of 1, 2, and 4 μg/ml (Fig. 3D). As such, iPSCs-KCs-Exos can enhance the migration of endothelial cells and keratinocytes migration without altering the proliferative activity of these cells.

### miR-762 is enriched within iPSCs-KCs-Eoxs and serves as a key mediator of iPSCs-KCs-Exos-induced migration of keratinocytes and endothelial cells

**Fig. 4:**
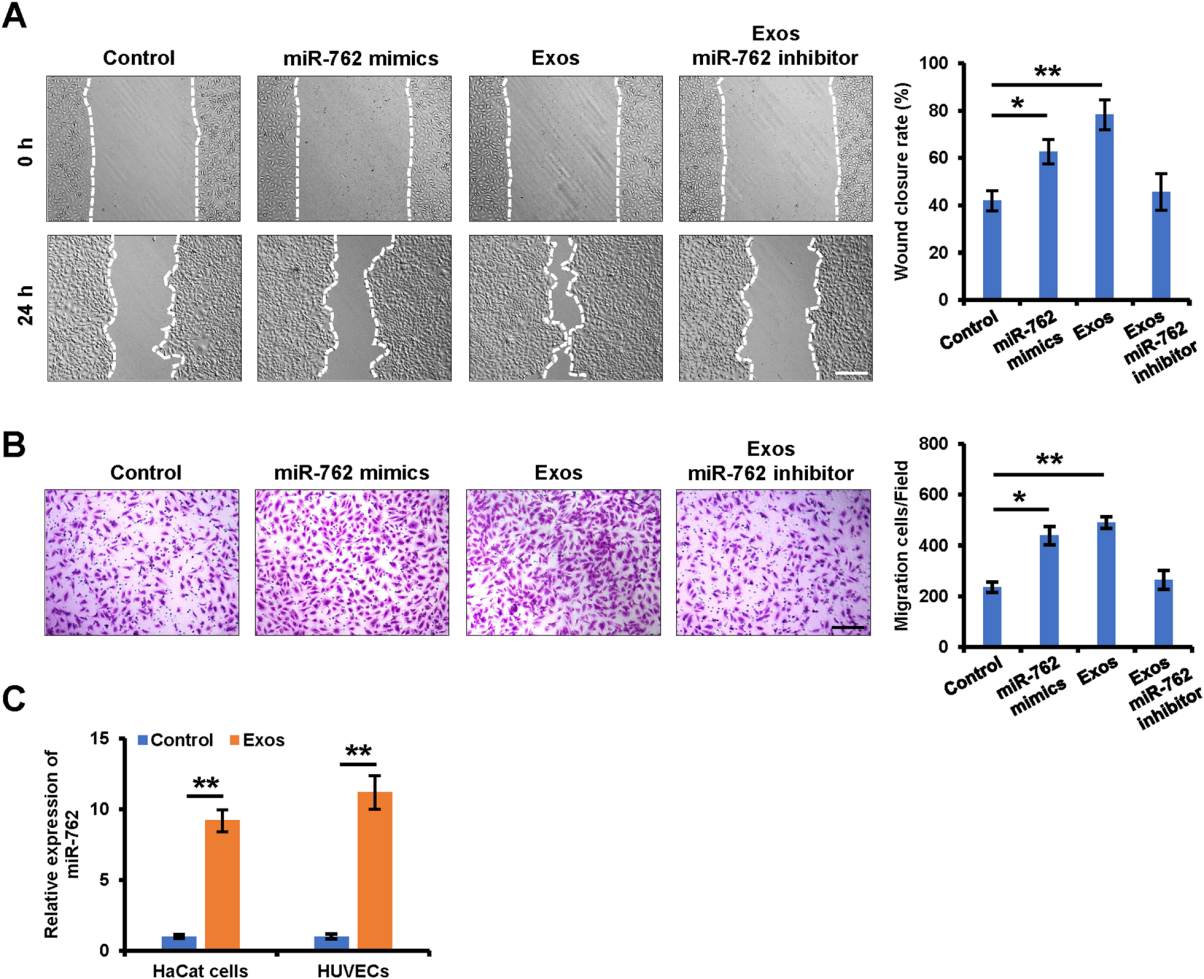
Mir-762 is a key mediator of iPSCs-KCs-Exos induced migration of keratinocytes and endothelial cells. **A** Representative images of the wound healing assay of keratinocytes in each group (left panel). Scale bar = 100 μm. Quantitative analysis of the wound healing rates in each group (right panel). **B** Images of migrated HUVECs in each group. Scale bar, 200 μm. Quantitative analysis of the migrated cells (right panel). Scale bar, 200 μm. **C** qRT–PCR analysis of miR-762 expression in HaCaT cells and HUVECs which were treated with iPSCs-KCs-Exos (2 μg/mL) for 24 h. *P < 0.05, **P < 0.01

While microRNAs (miRNAs) account for a relatively small fraction of the total content within extracellular vesicles, they have nonetheless been shown to be important modulators of cellular function [24]. To test the potential relevance of such miRNAs in the context of iPSCs-KCs-Exos-induced migratory activity and wound healing, miRNA microarray analysis was conducted using these exosomes. The result showed that miR-762 was the most abundant miRNA among these detected miRNAs (Table 1). Thus, we focused on exosomal miR-762 for the next investigation. In a wound healing assay with HaCaT cells, the overexpression of miR-762 resulted in enhanced wound closure rates at 24 h post-wounding (Fig. 4A). To further confirm the functional importance of this miRNA, iPSCs-KCs-Exos-treated HaCaT cells were treated with a specific inhibitor of miR-762, which attenuated this enhanced migratory activity (Fig. 4A). The same effect was also observed in HUVECs (Fig. 4B). In addition, the expression of miR-762 in HaCaT cells and HUVECs was indeed significantly elevated at 48 h post-iPSCs-KCs-Exos treatment, confirming the ability of these exosomes to deliver miR-762 to recipient cells (Fig. 4C). These data thus suggest that miR-762 is an important mediator of iPSCs-KCs-Exos-induced migration of keratinocyte and endothelial cells in vitro*.*

**Table 1:** Highly expressed miRNAs in iPSCs-KCs-Exos

Gene ID	Name	Read count
MIMAT0010313	hsa-miR-762	115,546
MIMAT0000266	hsa-miR-205-5p	114,148
MIMAT0000067	hsa-let-7f-5p	91,099
MIMAT0000069	hsa-miR-16-5p	51,004
MIMAT0000086	hsa-miR-29a-3p	28,959
MIMAT0000278	hsa-miR-221-3p	24,733
MIMAT0000415	hsa-let-7i-5p	22,310
MIMAT0000062	hsa-let-7a-5p	14,970
MIMAT0000063	hsa-let-7b-5p	13,934
MIMAT0005898	hsa-miR-1246	13,716
MIMAT0000732	hsa-miR-378a-3p	13,533
MIMAT0000076	hsa-miR-21-5p	13,026
MIMAT0000259	hsa-miR-182-5p	12,760
MIMAT0000281	hsa-miR-224-5p	11,130
MIMAT0000083	hsa-miR-26b-5p	10,781
MIMAT0000226	hsa-miR-196a-5p	9398
MIMAT0000764	hsa-miR-339-5p	9122
MIMAT0000078	hsa-miR-23a-3p	8538
MIMAT0049032	hsa-miR-12136	8013
MIMAT0000100	hsa-miR-29b-3p	6287

### miR-762 targets PML to regulate the migratory activity of keratinocytes and endothelial cells through ITGB1

**Fig. 5:**
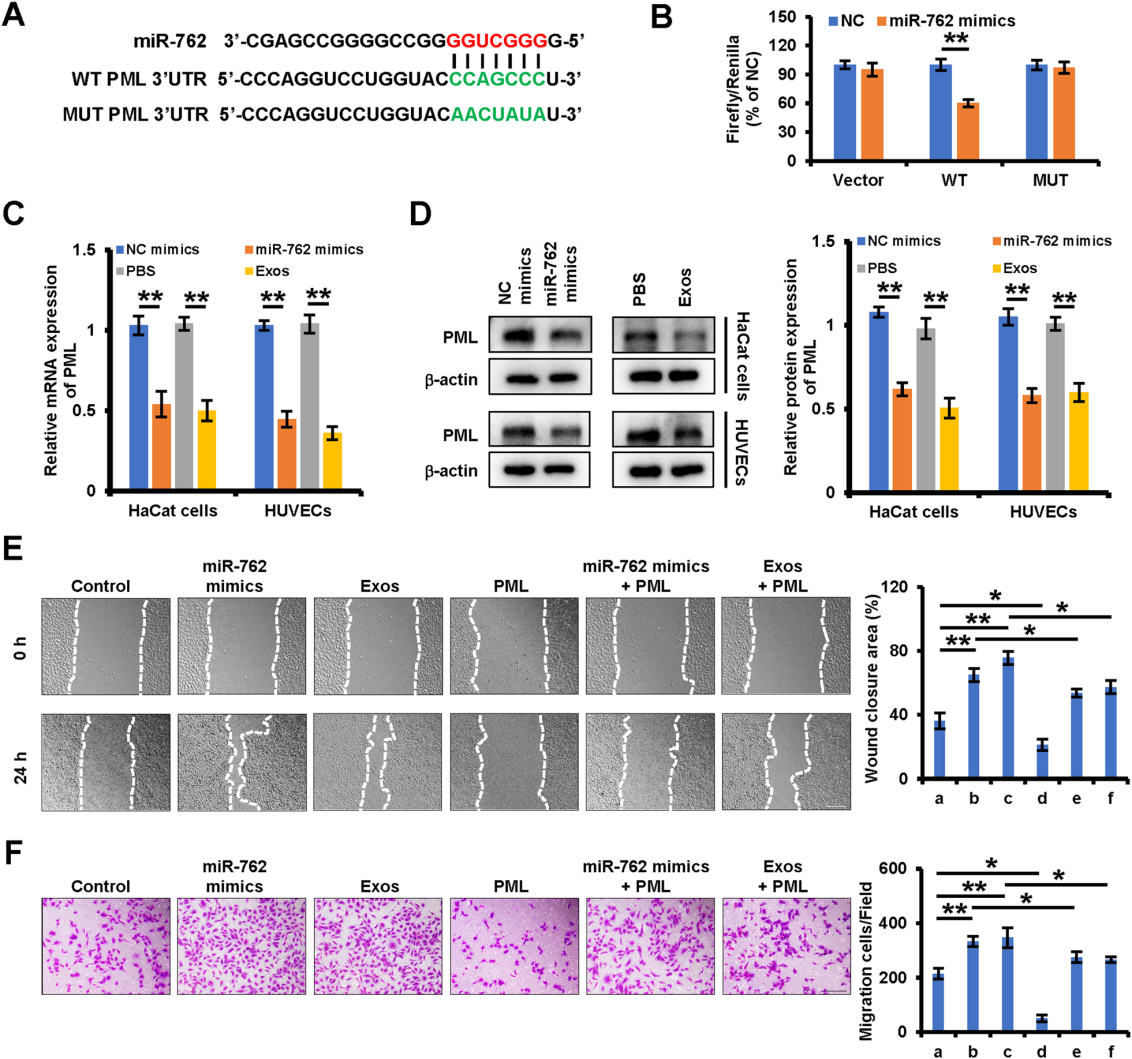
miR-762 and iPSCs-KCs-Exos promote migration of keratinocyte and endothelial cells by targeting PML. **A** Predicted miR-762 target sequences in PML 3’-UTR and the mutated nucleotides in the 3′UTR of PML. **B** The relative luciferase activity in 293 T cells with psiCHECK-PML-wt-3′UTR (WT) or psiCHECK-PML-mut-3’UTR (MUT) and miR-762 mimics co-transfection was evaluated by luciferase reporter assay. **C**, **D** qRT–PCR and Western blot analysis of PML expression in HaCaT cells and HUVECs which were treated with control, miR-762 mimics and iPSCs-KCs-Exos (Exos). **E** Representative images of the wound healing assay and quantitative analysis of the wound healing rates of HaCaT cells, which were treated with **a** control, **b** miR-762 mimics, **c** iPSCs-KCs-Exos (Exos), d pcDNA3.1-PML (PML), **e** miR-762 mimic plus pcDNA3.1-PML (PML), **f** iPSCs-KCs-Exos (Exos) plus pcDNA3.1-PML (PML). Scale bar, 200 μm. **F** Images of migrated HUVECs and quantitative analysis of the migrated cells, which were treated with **a** control, **b** miR-762 mimics, **c** iPSCs-KCs-Exos (Exos), d pcDNA3.1-PML (PML), **e** miR-762 mimic plus pcDNA3.1-PML (PML),** f** iPSCs-KCs-Exos (Exos) plus pcDNA3.1-PML (PML). Scale bar, 200 μm. *P < 0.05, **P < 0.01

To further clarify the regulatory role of iPSCs-KCs-Exos-derived miR-762 in the context of wound healing, potential target genes for this miRNA were next identified using the Targetscan, miRDB, and microRNA.org databases. This approach identified PML as a putative target of this miRNA. To confirm this prediction, luciferase reporter constructs containing wild-type (WT) or mutated (MUT) versions of the putative miR-762 binding site located within the PML 3’-untranslated region (UTR) were prepared (Fig. 5A). When 293 T cells were transfected with these reporters and miR-762 mimics, this miRNA was confirmed to suppress the activity of the WT but not the MUT reporter, confirming the predicted regulatory relationship (Fig. 5B). Moreover, mRNA and protein levels of PML were significantly lower in HaCaT cells and HUVECs following miR-762 mimic or iPSCs-KCs-Exos treatment (Fig. 5C, D). PML is thus a direct miR-762 target gene. To further examine the link between PML downregulation and the effects of miR-762 and iPSCs-KCs-Exos on the migration of keratinocytes and endothelial cells, the PML was overexpressed with the pcDNA3.1 vector. As shown in Fig. 5E, overexpression of PML inhibited the migration of HaCaT cells and could restrain the cell migration that was promoted by miR-762 mimics or iPSCs-KCs-Exos. Similar results were also observed in HUVECs (Fig. 5F). Taken together, the above results suggest that miR-762 and iPSCs-KCs-Exos induced acceleration of keratinocytes and endothelial cells are mediated at least partly by inhibiting PML expression.

### PML-regulated ITGB1 is involved in the promotion of keratinocytes and endothelial cells migration by miR-762 and iPSCs-KCs-Exos

**Fig. 6:**
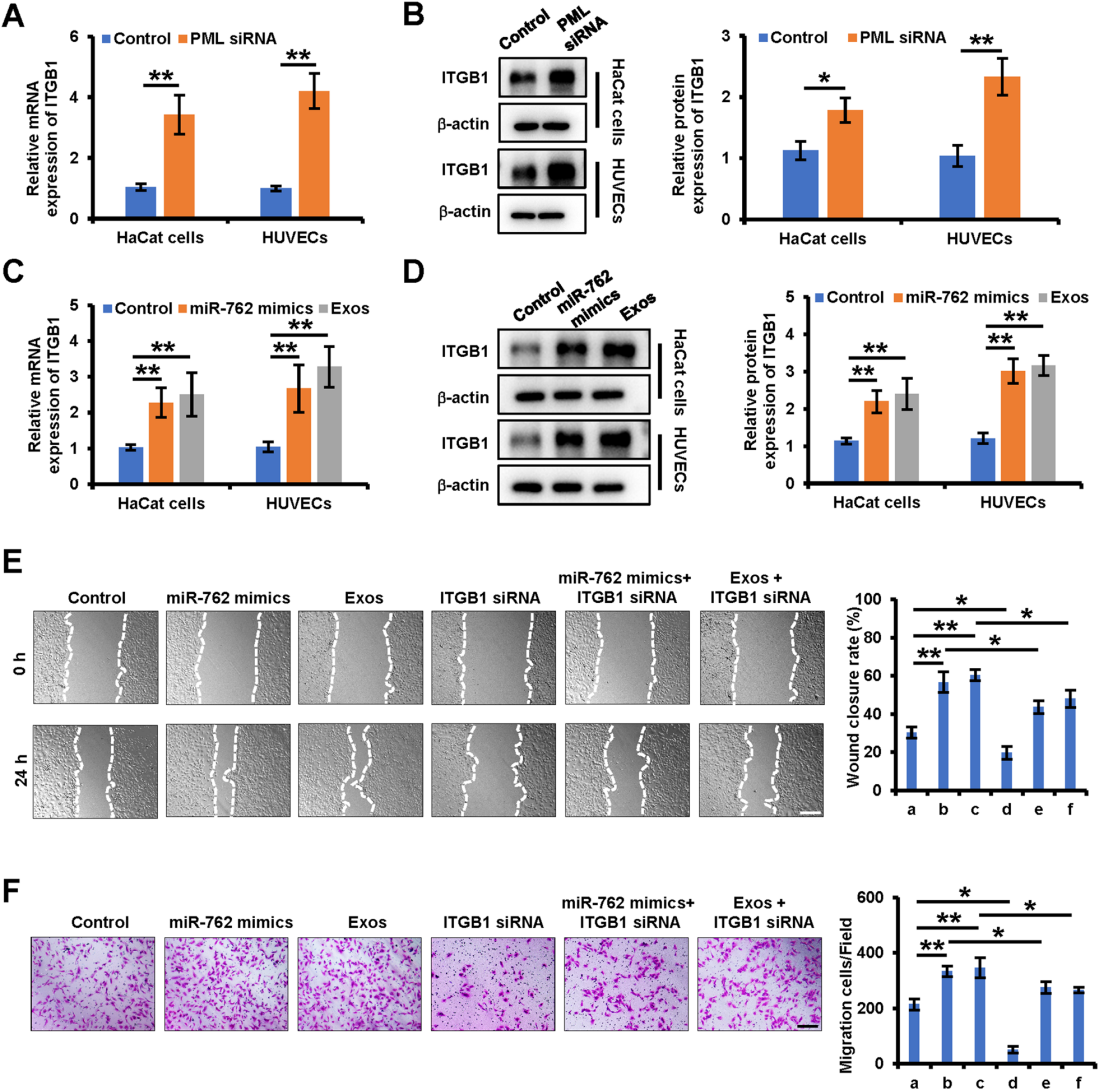
PML-regulated ITGB1 is involved in the promotion of keratinocytes and endothelial cell migration by iPSCs-KCs-Exos. **A**, **B** qRT–PCR and Western blot analysis of ITGB1 expression in HaCaT cells and HUVECs which were treated with control siRNA or PML siRNA. B Western blot analysis of protein expression of ITGB1 in each group. **C**, **D** qRT–PCR and Western blot analysis of ITGB1expression in HaCaT cells and HUVECs which were treated with control, miR-762 mimics, iPSCs-KCs-Exos. **E** Representative images of the wound healing assay and quantitative analysis of the wound healing rates of HaCaT cells, which were treated with **a** control, **b** miR-762 mimics, **c** iPSCs-KCs-Exos (Exos), d pcDNA3.1-ITGB1 (ITGB1), **e** miR-762 mimic plus pcDNA3.1- ITGB1 (ITGB1),** f** iPSCs-KCs-Exos (Exos) plus pcDNA3.1- ITGB1 (ITGB1). Scale bar, 200 μm. **F** Images of migrated HUVECs and quantitative analysis of the migrated cells, which were treated with **a** control, **b** miR-762 mimics, **c** iPSCs-KCs-Exos (Exos), d pcDNA3.1-ITGB1 (ITGB1), **e** miR-762 mimic plus pcDNA3.1- ITGB1 (ITGB1),** f** iPSCs-KCs-Exos (Exos) plus pcDNA3.1- ITGB1 (ITGB1). Scale bar, 200 μm. *P < 0.05, **P < 0.01

Reineke et al. previously demonstrated that ITGB1 was responsible for mediating PML-induced changes in migratory activity [25]. In this study, we examined the effects of PML knockdown on ITGB1 expression by siRNA. When PML expression decreased, we observed an increase in ITGB1 mRNA and protein expression in keratinocytes and endothelial cells (Fig. 6A, B). We further found that the expression of ITGB1 also increased after treatment with miR-762 mimic or iPSCs-KCs-Eoxs (Fig. 6C, D). Moreover, siRNA-mediated ITGB1 knockdown ablated the miR-762 mimic, and iPSCs-KCs-Eoxs induced migration of HaCaT cells and HUVECs (Fig. 6E, F). Together, these data suggest that miR-762 can target PML to enhance the migration of keratinocyte and endothelial cells through a mechanism involving ITGB1.

## Discussion

Herein, we found that iPSCs-KCs-Exos treatment was sufficient to promote keratinocyte migration and thereby accelerate the healing of deep second-degree burn wounds in mice. We additionally determined that the most abundant miRNA within these exosomes, miR-762, was a key mediator of this enhanced migratory effect owing to its ability to target PML.

In recent years, a variety of bioactive materials have been developed, which play a role in activating platelets, regulating the chemotaxis of immune cells, and accelerating cell migration and proliferation, respectively, according to the cellular and molecular mechanisms at each stage of wound healing [26]. For example, Li et al. designed an injectable nanocomposite by inserting quaternized carboxymethyl chitosan into the middle layer of organic rectorite, which allowed natural clay to combine with blood cells to achieve hemostasis [27]. A modular hydrogel consisting of a glycosaminoglycan heparin derivative and a star-shaped polyethylene glycol has been designed to maximize the sequestration of chemokines and thus reduce the migration activity of neutrophils and monocytes to the wound area [28]. A type of electrospinning nanofiber scaffold containing nagelschmidtite (NAGEL, Ca7P2Si2O16) has been shown to activate epithelial-mesenchymal transition and endothelial-mesenchymal transition, thereby promoting the process of epithelialization [29]. In addition, exosomes have also been proved to be a kind of biomaterial with great potential for wound therapy [30].

In the process of wound healing, keratinocytes, fibroblasts, and endothelial cells are the main cell populations involved in re-epithelialization and granulation of tissue construction [31]. Therefore, communication between these cells plays a key role in skin repair. This network is likely regulated by cell-derived exosomes, which carry a range of macromolecular cargos that can be delivered to target cells, enabling them to regulate recipient cell responses in a variety of complex manners [32–35]. Both HaCaT cells and primary keratinocytes can release exosomes and other extracellular vesicles [36, 37]. In their deep sequencing analysis of microRNAs within keratinocyte-derived extracellular vesicles, Than et al. found that the most enriched miRNAs found therein were linked to key physiological processes such as fibroblast migration and dermal-epithermal wound healing, suggesting a role for these vesicles in the context of cutaneous wound healing [21].

This study was designed to explore the ability of iPSC-KC-derived exosomes to regulate the process of burn wound healing. We successfully generated and characterized iPSCs-KCs-Exos, which were of the expected size and exhibited characteristic morphological features. Cutaneous wound healing processes are complicated and necessitate interplay between inflammatory, angiogenic, remodeling, proliferative, epithelialization, and fibrotic processes [38]. Histological analyses revealed that increases in re-epithelialization and capillary density were evident in wound sites following iPSCs-KCs-Exos treatment, but collagen formation was not affected. Thus, we then further examined the effects of these exosomes on endothelial cells and keratinocytes, which are the key mediators of angiogenesis and epithelialization, respectively. These analyses revealed that iPSCs-KCs-Exos significantly enhanced the migratory activity of these cells without impacting their proliferative activity. As such, the beneficial impact of these exosomes in the context of cutaneous wound healing may be primarily attributable to their ability to promote keratinocyte and endothelial cell migration.

Exosomes contain a variety of miRNAs, mRNAs, lipids, and proteins that are compiled within the ExoCarta database [14, 15]. Of these, miRNAs are the best-studied exosomal cargos owing to their ability to regulate target mRNA expression. Herein, we detected many miRNAs that were highly abundant within iPSCs-KCs-Exos, of which miR-762 was detected at the highest levels. Moreover, we found that miR-762 was able to promote endothelial cell and keratinocyte migration in a manner similar to iPSCs-KCs-Exos treatment, suggesting that it is likely a primary mediator of the phenotypic effects-KCs-Exos in the context of wound healing. In prior research, miR-762 has been found to regulate a range of target genes in different contexts. The upregulation of miR-762 in non-small cell lung cancer, bladder cancer, breast cancer, and nasopharyngeal carcinoma has been linked to enhanced tumor cell invasivity, migration, and proliferation [39–42]. However, there have been no prior studies regarding the role of this specific miRNA in keratinocytes or endothelial cells. In line with these past findings, we observed a pro-migratory role for miR-762 in both of these cell types.

Herein, we identified promyelocytic leukemia (PML) as a novel miR-762 target gene associated with the ability of this miRNA to regulate endothelial cell and keratinocyte migration. PML plays a role in many important cellular processes, including tumor suppression, apoptosis, transcriptional regulation, DNA damage response, senescence, and viral defense mechanisms [43–45]. Herein, we confirmed that PML was a direct miR-762 target, and we found that overexpressing PML was sufficient to reverse the beneficial impact of miR-762 on cellular migration. In prior reports, the ability of PML to suppress the migration of MDA-MB-231 cells was attributed to its ability to promote ITGB1 downregulation [25]. ITGB1 is important for attachment to and integrity of cell‐extracellular matrix (BME) interaction by mediating binding to BME components, and they are generally required for migration of keratinocytes and endothelial cells [46, 47]. We thus explored the link between ITGB1 and this miR-762/PML regulatory axis, leading us to conclude that PML overexpression suppressed ITGB1 expression while miR-762 was able to reverse this effect. We further confirmed a role for ITGB1 in the miR-762/PML-mediated migratory phenotypes observed in this experimental system. However, as miRNAs target multiple genes, these results do not offer any insight into the ability of miR-762 to regulate other signaling pathways within keratinocytes. Moreover, while PML and ITGB1 clearly play a role in this process, further work is required to elucidate the underlying mechanisms.

In conclusion, these results indicate that iPSCs-KCs-Exos treatments can significantly improve burn wound healing outcomes, highlighting novel approaches to expediting cutaneous wound healing and providing a foundation for future research efforts. There are also some limitations of this study. We only tested the effect of iPSCs-KCs-Exos on keratinocytes and endothelial cells, so more research is needed in the future to explore the possibility of iPSCs-KCs-Exos affecting other different cell types during all phases of wound healing. For clinical application, a more efficient and economical generation of keratinocytes from iPSCs needs to be developed. As with other types of exosomes, advances in large-scale production technology and quality control of exosomes will enhance the use of iPSCs-KCs-Exos in wound healing applications in the near future.

## Data Availability

The authors agree to share data and materials related to this manuscript.

## Abbreviations

iPSCs: Induced pluripotent stem cells
iPSCs-KCs: IPSCs-derived keratinocytes
iPSCs-KCs-Exos: IPSCs-KCs-derived exosomes
PML: Promyelocytic leukemia
ITGB1: Integrin beta1
UM: Unconditioned medium
RA: Retinoic acid
K-DSFM: Keratinocyte defined serum-free Medium
DMEM: Dulbecco’s modified eagle’s medium
HUVECs: Human umbilical vein endothelial cells
PBS: Phosphate-buffered saline
TEM: Transmission electron microscopy
NTA: Nanoparticle tracking analysis
H&E: Hematoxylin and eosin
DAPI: 4,6-Diamidino-2-phenylindole
EdU: 5-Ethynyl-2′-deoxyuridine
qRT–PCR: Quantitative real-time polymerase chain reaction
K14: Cytokeratin-14
miRNAs: MicroRNAs
BME: Cell‐extracellular matrix
